# Necrotizing Autoimmune Myopathy: A Case Report on Statin-Induced Rhabdomyolysis

**DOI:** 10.7759/cureus.49065

**Published:** 2023-11-19

**Authors:** Faryal Altaf, Vedangkumar Bhatt, Abeer Qasim, Zaheer A Qureshi, Vijil Rajan, Sarah Moore, Rene Elkin

**Affiliations:** 1 Department of Internal Medicine, Continental Medical College, Lahore, PAK; 2 Department of Internal Medicine, BronxCare Health System, New York, USA; 3 Department of Internal Medicine, The Frank H. Netter M.D. School of Medicine at Quinnipiac University, Bridgeport, USA; 4 Department of Internal Medicine, St. Vincent's Medical Center, Bridgeport, USA; 5 Department of Internal Medicine, American University of the Caribbean School of Medicine, Cupecoy, SXM; 6 Department of Neurology, BronxCare Health System, New York, USA

**Keywords:** general internal medicine, medicine, atorvastatin, statin, rheumatology, creatinine kinase, rhabdomyolysis, myopathy, anti-hmg coa antibody, necrotizing autoimmune

## Abstract

Statin-induced necrotizing myopathy (SINM) is an uncommon but severe complication associated with statin medication. SINM can develop at any point after a person starts taking steroids. It is now being acknowledged as a component of the broader category of "statin-induced myopathy." Like other immune-mediated necrotizing muscle diseases, statin-induced myositis is identified by weakness in proximal muscles, increased serum creatine kinase (CK) levels, and, in some cases, dysphagia and respiratory distress. In addition, there is evidence of muscle cell damage when examined under a microscope, occurring with minimal or no infiltration of inflammatory cells. Diagnosing SINM promptly is frequently challenging due to its unpredictable development over time, with symptoms sometimes emerging many years after the initial exposure to statins. One distinctive characteristic of SINM is the continued presence of muscle inflammation and elevated CK levels even after discontinuing statin treatment. Currently, no clinical trials are available to guide how to manage statin-induced immune-mediated necrotizing myopathy (IMNM). Here, we present a case of a 42-year-old woman diagnosed with SINM and was found to have persistently elevated CPK despite discontinuation of statins. Our case also suggests that intravenous (IV) immunoglobins and steroids are an effective and well-tolerated alternative to immunosuppressants.

## Introduction

Immune-mediated necrotizing myopathy (IMNM) has been seen in adults and children, and the incidence of this myopathy has been increasing in recent years [1]. Females are most affected by this myopathy, as seen in other autoimmune diseases [1,2]. There are two main types of IMNM: the first is anti-signal recognition particle (SRP)-positive IMNP and the second one is anti-3-hydroxy-3-methyl-glutaryl-coenzyme A reductase (HMGCR) antibody-positive IMNM [2]. The HMGCR antibody has been observed in people exposed to statins but in people without statins [2,3]. Still, HMG CoA-mediated necrotizing myopathy (NM) is rare among statin users, with an approximate incidence of two to three in every 100,000 patients using statins [3]. Atorvastatin has been seen as the causative factor in most cases [1]. There are a few subtypes of IMNM, namely, seronegative IMNM and cancer-associated IMNM; they are associated with different auto-antibodies, such as anti-Ro52, anti-PM-Scl, anti-mitochondrial (AMA), anti-U1-RNP, or an antisynthetase antibody, or they may be unaccompanied by any known autoantibodies [4]. Here, we present a case of a 42-year-old female with no medical history of any autoimmune disease found to have IMNM caused by statin and was found to be positive for the HMGCR antibody.

## Case presentation

A 42-year-old female with a medical history of hypertension, obesity, and recently diagnosed hyperlipidemia (seven months ago) presented due to complaints of muscle aches, purpura on her left arm, progressive odynophagia, and tongue swelling for the past three weeks. Past surgical history was unremarkable, and family history was significant for type II diabetes mellitus in the mother. On presentation, the patient was hemodynamically stable with a heart rate of 92 beats per minute, temperature of 98 F, and blood pressure of 119/69 mmHg. The patient showed symptoms of proximal muscle weakness, including the ability to stand up from a chair using hands. Her home medications include semaglutide and newly started atorvastatin. She was started on atorvastatin 40 mg seven months ago. On examination, the rash was purpuric, and the patient was noted to have weakness in the proximal and distal muscles in the arms and legs and mild tongue swelling; the rest of the examination was unremarkable. Her laboratory investigations were significant for transaminitis with elevated aspartate aminotransferase (AST) of 1088 unit/L and alanine aminotransferase (ALT) of 721 unit/L with normal bilirubin and elevated creatinine kinase (CK) of 22,000 U/L indicative of rhabdomyolysis, for which she was started on intravenous (IV) crystalloid fluid. Her initial laboratory results are shown in Table 1.

**Table 1:** Initial laboratory tests MCV: mean corpuscular volume, INR: international normalized ratio, APTT: activated partial thromboplastin time, ALT: alanine transaminase, AST: aspartate transaminase, GGT: gamma-glutamyl transpeptidase

Laboratory test	Patient values	Reference values
Red blood cell (RBC)	5.36 MIL/ul	4.00-5.20 MIL/ul
Hemoglobin (Hb)	16.2 g/dl	12.0-16.0 g/dl
MCV	86.7 fL	80.0-96.0 fL
Platelet count	298 k/ul	150-400 k/ul
Neutrophil count	7.3 k/ul	1.5-8.0 k/ul
Lymphocyte count	2 k/ul	1.0-4.8 k/ul
Prothrombin time (PT)	14.5 s	9.9-13.3 second(s)
INR	1.24	.85-1.14
APTT	32 s	27.2-39.6 second(s)
Sodium	135 mEq/L	135-145 mEq/L
Potassium	4.1 mEq/L	3.5-5.0 mEq/L
Creatinine	0.5 mg/dl	0.5-1.5 mg/dl
Calcium ionized	1.15 mmoles/L	1.15-1.29 mmoles/L
Magnesium	2.3	1.5-2.7 mg/dl
Phosphorous	4.2	2.5-4.5 mg/dl
ALT	721 unit/L	5-40 unit/L
AST	1088 unit/L	9-36 unit/L
GGT	189 unit/L	8-54 unit/L
Albumin, serum	4.5 g/dl	3.2-4.8 g/dl
Total protein serum	8.1 g/dl	6.0-8.5 g/dl

**Figure 1:**
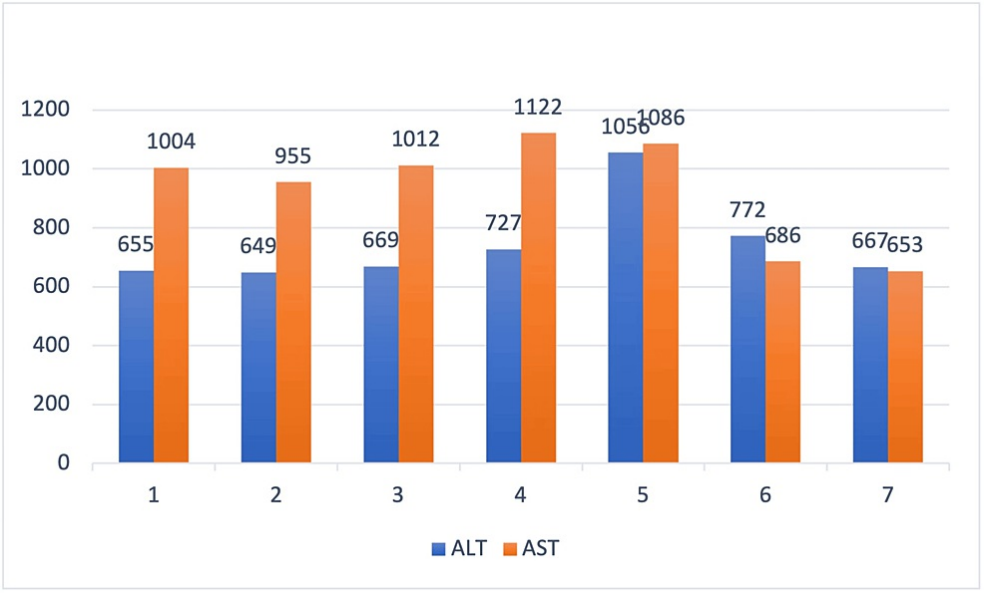
ALT:AST trend x-axis shows days in the hospital; y-axis shows laboratory value in U/L. ALT: alanine aminotransferase, AST: aspartate aminotransferase

**Figure 2:**
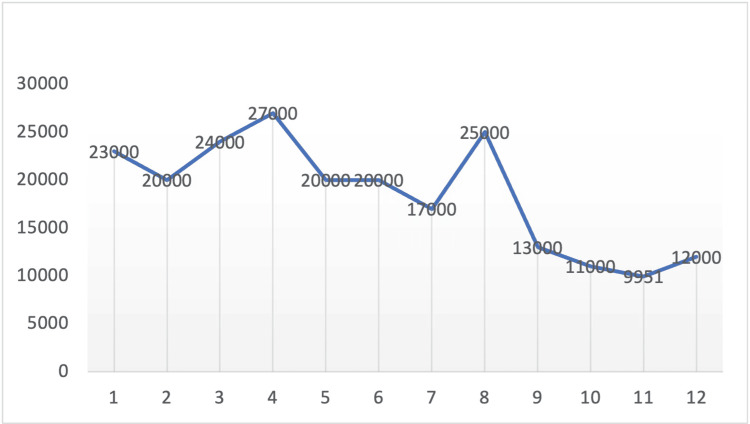
Creatinine kinase (CK) trend x-axis shows days in the hospital; y-axis shows the laboratory results of creatinine kinase (CK) in U/L (units per liter).

**Figure 3:**
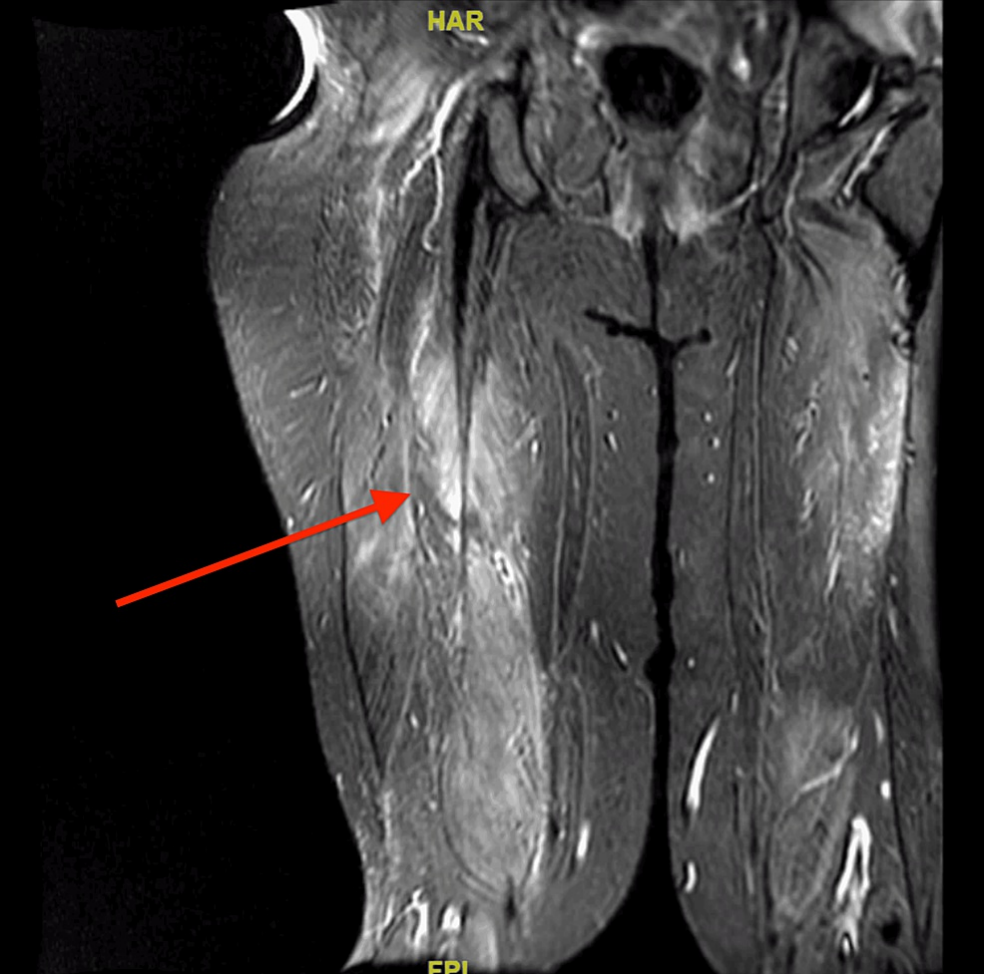
MRI of bilateral lower extremities showing fairly symmetric moderate bilateral myositis throughout bilateral thigh The red arrow shows myositis.

Moreover, atorvastatin was held due to transaminitis and rhabdomyolysis. The rest of the findings, including autoimmune workup, are shown in Table 2. She also had a negative hepatitis panel. The patient also complained of dysphagia and underwent a flexible fiberoptic laryngopharyngoscopy (FEES), which was normal. Due to persistent muscle weakness and pain and up-trending LFTs and CPK, as shown in Figures 1 and 2, she underwent an MRI of her lower extremities, showing multifocal areas of fairly symmetric appearing moderate intramuscular edema scattered throughout the bilateral lower extremities. MRI findings were consistent with multifocal areas of bilateral myositis, as shown in Figure 3: myositis panel and anti-HMG CoA. A reductase antibody was sent, which was strongly positive, as shown in Table 3. A final diagnosis of HMGCR antibody-positive necrotizing myositis caused by statin was made, and she was started on prednisone 60 mg and immunoglobulin (IVIG) 2 g/kg infusion for five days with a plan to continue IVIG once a month for six months. Soon after initiating the above regimen, her CPK levels started to trend down, as shown in Figure 2. The patient's symptoms improved, and she was discharged with a plan to follow rheumatology in the outpatient clinic.

**Table 2:** Antibody panel results ANA: antinuclear antibody, AMA: anti-mitochondrial autoantibodies, CRP: C-reactive protein, EBV: Epstein-Barr virus, SMA: smooth-muscle antibodies, SS: Sjögren's syndrome, TSH: thyroid-stimulating hormone, CK: creatine kinase, UIBC: unsaturated iron-binding capacity

Laboratory test	Patient values	Reference values
ANA	Negative	Negative
AMA	Negative	Negative
CRP	18.76 mg/L	<=5.00 mg/L
EBV-VCA IgM	<36 U/ml	<36 U/ml
EBV-VCA IgG	>750.00 U/ml	<18 U/ml
SMA	negative	Negative
SS-A AB	>8 (positive)	<1 Negative
SS-B Ab	<1 (negative)	<1 Negative
Anti-smooth muscle	Negative	Negative
TSH	4.88 mIU/L	0.4-4.50 mIU/L
CK	20.000 unit/L	20-200 Unit/L
Ceruloplasmin, serum	33 mg/dl	18-53 mg/dl
Liver kidney microsomal assay	<20 U	<=20.0 U
Ferritin	144 ng/mL	13.0-150.0 ng/mL
UIBC	255 ug/dl	112-346 ug/dl
Iron, serum	56 ug/dl	65-175 ug/dl

**Table 3:** Myositis-specific antibodies RNP: ribonucleoprotein, HMG-CoA: hydroxymethylglutaryl coenzyme A

Laboratory test	Patient values	Reference values
Cryoglobulin	Not detected	None detected
Anti-RNP	<1 negative	<1 negative
Aldolase	309 U/L	<=8.1 U/L
HMG-CoA reductase antibody	Positive	<20.0 CU

## Discussion

SINM is a part of a heterogeneous family of inflammatory diseases, which presents a diagnostic challenge because of its broad clinical presentation [5]. SINM is known to develop in two to three patients for every 100,000 statin users, making it a relatively rare complication of the drug [6]. There is no evidence of statin use impacting the initiation or severity of this disease, as it has been recorded to occur even years after the initial exposure [7]. SINM does not require the present use of statins but can occur in past users as well; interestingly, it is known to manifest in individuals exposed to statin-like compounds found in red yeast rice or oyster mushrooms [8]. In this case, the patient presented symptoms seven months after the initial prescription of atorvastatin 40 mg; late onset of symptoms causes difficulty in identifying triggering factors. 

The clinical manifestations of SINM are consistent with inflammatory myopathies, which includes muscle weakness, elevated CK levels, diagnostic antibodies, and increased serum level of muscle enzymes [9]. The patient was presented with progressive generalized muscle weakness for three to four weeks, and it was sufficiently severe that she faced significant difficulty in ambulation. CK levels in SINM have a typical range of 10 and 100 times the upper limit of normality, and this patient’s initial evaluation showed a serum CK level of 23,625 U/L [10]. A further indication of myositis pathology came from elevated muscle enzymes, specifically the patient’s aldolase level being 70.8 ug/L (normal range 3.3-10.3 ug/L). Furthermore, dysphagia is known to occur as a part of the initial presentation due to the inflammatory effects on pharyngeal muscles, causing reduced contractility, hypomotility of the esophagus, and cricopharyngeal dysfunction [11]. Consistent with the literature, this patient also presented with dysphagia with continued difficulty swallowing and choking sensation. During the hospital stay, her diet was managed by a dysphagia diet. 

Statins have been known to cause liver toxicity with a frequency of one in 100,000 patient years and have been identified to trigger elevated amino transaminases in 3% of the patients’ prescribed statins [12]. Although the exact mechanism is not entirely understood, recent studies have suggested that statin use increases reactive oxygen species, eventually causing lipid peroxidation and cytotoxicity [13]. This patient persistently had elevated aminotransferases, with the initial AST and ALT being 1004 U/L and 655 U/L, respectively. The transaminitis present in this patient endured for an extended period, which eventually improved after discontinuing statins and initiating SINM therapy. Although infrequent, liver function tests should be checked before prescribing statins, and symptomatic follow-up should involve evaluating the lipid profile and LFTs [14]. Discontinuation of statins is only required if the liver enzymes are elevated three times the upper limit of the normal range [15].

Diagnosis of SINM requires the detection of anti-HMGCR antibodies. One of the proposed mechanisms of SINM suggests an increased expression of HMGCR post-initiation of statins, leading to an autoimmune pathology [16]. This persists even after the discontinuation of the medication, and thus, immunosuppressive therapy is required [16]. The autoimmune process is suspected to be caused by anti-HMGCR antibodies cross-reacting with an antigen associated with myositis [17]. This patient was found to have an anti-HMGCR antibody level of 145 CU, which suggests a strong positive result consistent with this pathology. Interestingly, there have been rare cases where the anti-HMGCR antibody test returned positive, even in those who had never started statin therapy [18]. Regardless, the anti-HMGCR antibody test is clinically significant, with a recorded sensitivity of 94.4% and a specificity of 99.3%. Thus, a positive result indicates a high likelihood of an autoimmune myopathy involving statins [19]. 

The treatment and management for SINM patients involves the abrupt discontinuation of statins and immunosuppressive therapy [20]. However, there are no gold-standard guidelines as most of these treatment techniques recommended are accumulated information from case series, anecdotal experience, and observational studies of inflammatory myopathies [21]. The typical course of treatment for SINM is induction of steroids and maintenance with immunosuppressive therapy. Immunosuppressants, such as methotrexate, azathioprine, and mycophenolate mofetil, have improved clinical outcomes. Furthermore, treatment-resistant patients may require IVIG or rituximab therapy [22]. IVIG is the first-line treatment for diabetic patients and can also be used as monotherapy [23]. This patient was started on prednisone 60 mg and IVIG 130 mg; this resulted in an improvement in clinical symptoms along with a significant reduction in her CK levels. The patient noted an improvement in muscle weakness and does not endorse dysphagia symptoms; further follow-ups and management will be required to decrease the risk of exacerbation. Long-term management for SINM is also an option in patients with frequent disease recurrence [24]. The risk of relapse is considerably reduced with vigilant symptom monitoring and consistent medication adherence [25]. SINM prognosis is typically favorable. However, it is recommended for these patients to be screened for malignancy as there is a notable elevated risk after the age of 50 and during the initial three years of post-diagnosis [25].

Statins are divided into lipophilic and hydrophilic agents depending on their ability to pass deeply into the cell membrane, interacting with the surrounding acyl chains [26]. Lipophilic agents include simvastatin, fluvastatin, lovastatin, pitavastatin, and atorvastatin [26]. Hydrophilic agents include pravastatin and rosuvastatin [27,28]. It is a common belief that lipophilic statin increases myotoxicity because of its ability to diffuse in a non-selective way across the cell membrane of non-liver tissues as compared to the hydrophilic one [29,30]. For the abovementioned reason, hydrophilic statin should be preferred [30].

## Conclusions

Statin-induced IMNM is an infrequent yet severe condition linked to the usage of statin drugs. Although it is an uncommon adverse outcome of using statins, healthcare providers should remain vigilant and consider the possibility of this rare condition in patients experiencing muscle-related symptoms associated with statin use, especially if the symptoms persist despite discontinuing the medication. To diagnose necrotizing autoimmune myopathy (NAM), it is crucial to rule out more prevalent autoimmune myopathic disorders, metabolic issues, and neurological causes that can lead to weakness in the muscles around the hips and thighs or nerve-related problems. This condition should be considered as a potential diagnosis when dealing with ongoing muscle weakness and elevated CK levels in individuals who have been exposed to statins, especially if the symptoms persist even after stopping statin treatment. Additional research is necessary to determine who is susceptible to developing statin-induced NAM and to establish whether there are specific individuals who should altogether avoid statin therapy.
